# Whole-Genome Sequence for Methicillin-Resistant Staphylococcus aureus Strain ATCC BAA-1680

**DOI:** 10.1128/genomeA.00011-15

**Published:** 2015-03-12

**Authors:** Luke T. Daum, Violet V. Bumah, Daniela S. Masson-Meyers, Manjeet Khubbar, John D. Rodriguez, Gerald W. Fischer, Chukuka S. Enwemeka, Steve Gradus, Sanjib Bhattacharyya

**Affiliations:** a Longhorn Vaccines & Diagnostics, San Antonio, Texas, USA; b College of Health Sciences, University of Wisconsin-Milwaukee, Milwaukee, Wisconsin, USA; c City of Milwaukee Health Department, Milwaukee, Wisconsin, USA; d San Diego State University, San Diego, California, USA

## Abstract

We report here the whole-genome sequence of the USA300 strain of methicillin-resistant Staphylococcus aureus (MRSA), designated ATCC BAA-1680, and commonly referred to as community-associated MRSA (CA-MRSA). This clinical MRSA isolate is commercially available from the American Type Culture Collection (ATCC) and is widely utilized as a control strain for research applications and clinical diagnosis. The isolate was propagated in ATCC medium 18, tryptic soy agar, and has been utilized as a model S. aureus strain in several studies, including MRSA genetic analysis after irradiation with 470-nm blue light.

## GENOME ANNOUNCEMENT

Methicillin-resistant Staphylococcus aureus (MRSA) is a Gram-positive bacterium responsible for a variety of clinical manifestations and increasing cases of community-acquired infections (1). MRSA infection accounts for 44% of all hospital associated infections in the United States (2), and as many as 92% of persons hospitalized for MRSA have community-acquired (CA-MRSA), typically from the USA300 strain.

Whole-genome characterization has been performed for a number MRSA clinical and reference S. aureus strains (3, 4). This is the first report of a whole-genome sequence for an American Type Culture Collection (ATCC) strain designated ATCC BAA-1680 (referred to here as reference strains 25b and 26b). Additionally, we report whole-genome sequences containing synonymous and nonsynonymous nucleotide mutations of four MRSA ATCC BAA-1680 isolates, 27b, 29b, 31b, and 33b, after exposure to blue light (at 470 nm) irradiation at 3, 55, 110, and 110 J/cm^2^, respectively.

Total genomic DNA (gDNA) from culture grown MRSA reference isolates (25b and 26b) was obtained using the PrimeXtact total genomic extraction spin kit, as described earlier (5). For next-generation sequencing (NGS) library preparation, approximately 5 ng of input gDNA was processed using the Nextera XT sample prep kit (Illumina, San Diego, CA, USA). MiSeq NGS was performed according to the manufacturer’s instructions (Illumina) using the MiSeq reagent kit (version 3) with 600 cycles. Following fragmentation, end preparation, and sample tagging, the sequencer produced 6.2 and 6.3 million paired reads for ATCC BAA-1680 reference strains designated 25b and 26b, respectively, yielding appreciable coverage of ~200× for both strains. Assembly was performed using LaserGene DNAStar N-Gen version 12.1 (LaserGene, Inc., Madison, WI, USA). The strain 25b assembly resulted in a single contig totaling 2.3 billion bases with 6.2 million total reads (average read length, 376 bases), while the strain 26b assembly resulted in a single contig totaling 2.2 billion bases with 6.3 million total reads (average read length, 352 bases). Annotations were performed by the NCBI Prokaryotic Genomes Annotation Pipeline. The MRSA chromosome of reference strain 25b (accession no. CP010299) contains 2,937 genes, 2,845 coding sequences (CDSs), 16 rRNAs, and 52 tRNAs, while the chromosome of strain 26b (accession no. CP010298) contains 2,938 genes, 2,846 CDSs, 16 rRNAs, and 52 tRNAs.

The ATCC BAA-1680 strains 25b and 26b were used as controls for a larger study to investigate possible genetic effects underlying the antimicrobial effects of 470-nm blue light on MRSA and any genetic differences between irradiated and control MRSA strains upon whole-genome sequence comparisons. The effective antimicrobial properties that blue light irradiation has on MRSA may be attributed to mutational changes identified by whole-genome analysis and may facilitate a better understanding of the antimicrobial effect of blue light, an alternative armamentarium for combating MRSA infections (6).

### Nucleotide sequence accession numbers.

The whole-genome sequences of MRSA ATCC BAA-1680 control strains 25b and 26b were deposited in the DDBJ/EMBL/GenBank databases under the accession numbers CP010299 and CP010298, respectively. The whole-genome sequences of four MRSA ATCC BAA-1680 blue light-treated strains were deposited in the DDBJ/EMBL/GenBank databases under the following accession numbers: CP010300 (strain 27b [at 3 J/cm^2^]), CP010295 (strain 29b [at 55 J/cm^2^]), CP010296 (strain 31b [at 110 J/cm^2^]), and CP010297 (strain 33b [at 110 J/cm^2^]).
